# Teaching Clinical Reasoning in Health Care Professions Learners Using AI-Generated Script Concordance Tests: Mixed Methods Formative Evaluation

**DOI:** 10.2196/76618

**Published:** 2025-11-20

**Authors:** Alexandre Hudon, Véronique Phan, Bernard Charlin, René Wittmer

**Affiliations:** 1 Department of Psychiatry and Addictology, Faculty of Medicine, Université de Montréal, Pavillon Roger-Gaudry, 2900 Bd Édouard-Montpetit Local L-315, Montréal, QC, H3T 1J4, Canada, 1 514 343 6111; 2 Department of Psychiatry, Institut universitaire en santé mentale de Montréal, Montreal, QC, Canada; 3 Centre de recherche de l'Institut universitaire en santé mentale de Montréal, Montreal, QC, Canada; 4 Department of Psychiatry, Institut national de psychiatrie légale Philippe-Pinel, Montreal, QC, Canada; 5 Groupe Interdisciplinaire de recherche sur la cognition et le raisonnement professionnel (GIRCoPRo), Université de Montréal, Montreal, QC, Canada; 6 Department of Pediatrics, Faculty of Medicine, Université de Montréal, Montreal, QC, Canada; 7 Department of Pediatrics, Department of Pediatrics, Centre Hospitalier Universitaire Sainte-Justine, Montreal, QC, Canada; 8 Centre de pédagogie appliquée au sciences de la santé, Faculty of Medicine, Université de Montréal, Montreal, QC, Canada; 9 Department of Family Medicine and Emergency Medicine, Faculty of Medicine, Université de Montréal, Montreal, QC, Canada

**Keywords:** script concordance test, clinical reasoning, artificial intelligence, large language models, medical education, formative assessment, generative AI, expert simulation

## Abstract

**Background:**

The integration of artificial intelligence (AI) in medical education is evolving, offering new tools to enhance teaching and assessment. Among these, script concordance tests (SCTs) are well-suited to evaluate clinical reasoning in contexts of uncertainty. Traditionally, SCTs require expert panels for scoring and feedback, which can be resource-intensive. Recent advances in generative AI, particularly large language models (LLMs), suggest the possibility of replacing human experts with simulated ones, though this potential remains underexplored.

**Objective:**

This study aimed to evaluate whether LLMs can effectively simulate expert judgment in SCTs by using generative AI to author, score, and provide feedback for SCTs in cardiology and pneumology. A secondary objective was to assess students’ perceptions of the test’s difficulty and the pedagogical value of AI-generated feedback.

**Methods:**

A cross-sectional, mixed methods study was conducted with 25 second-year medical students who completed a 32-item SCT authored by ChatGPT-4o (OpenAI). Six LLMs (3 trained on the course material and 3 untrained) served as simulated experts to generate scoring keys and feedback. Students answered SCT questions, rated perceived difficulty, and selected the most helpful feedback explanation for each item. Quantitative analysis included scoring, difficulty ratings, and correlations between student and AI responses. Qualitative comments were thematically analyzed.

**Results:**

The average student score was 22.8 out of 32 (SD 1.6), with scores ranging from 19.75 to 26.75. Trained AI systems showed significantly higher concordance with student responses (ρ=0.64) than untrained models (ρ=0.41). AI-generated feedback was rated as most helpful in 62.5% of cases, especially when provided by trained models. The SCT demonstrated good internal consistency (Cronbach α=0.76), and students reported moderate perceived difficulty (mean 3.7, SD 1.1). Qualitative feedback highlighted appreciation for SCTs as reflective tools, while recommending clearer guidance on Likert-scale use and more contextual detail in vignettes.

**Conclusions:**

This is among the first studies to demonstrate that trained generative AI models can reliably simulate expert clinical reasoning within a script-concordance framework. The findings suggest that AI can both streamline SCT design and offer educationally valuable feedback without compromising authenticity. Future studies should explore longitudinal effects on learning and assess how hybrid models (human and AI) can optimize reasoning instruction in medical education.

## Introduction

One of the biggest challenges in medical education is evaluating clinical capabilities, especially higher-order abilities such as clinical reasoning and decision-making. Knowledge-based tests, such as multiple-choice questions, frequently fall short of capturing the subtleties of clinical reasoning in the face of diagnostic uncertainty [1]. By simulating clinical interactions, tools such as workplace-based assessments and Objective Structured Clinical Examinations try to assess clinical reasoning, but they come with a high logistical and human cost. An additional method for addressing thinking in clinical environments with unclear definitions is the script concordance test (SCT). In contrast to standard assessments, SCTs ask students to use a Likert-type scale to rate the impact of scenarios that are followed by additional clinical information on a postulated hypothesis. Concordance with a panel of expert responses serves as the basis for scoring, which reflects the variety and probabilistic nature of actual clinical practice [2,3]. SCTs are valuable, but creating and scoring them requires time and involves hiring several specialists who must give careful, consistent answers to a variety of questions.

The format of an SCT usually involves presenting a clinical vignette followed by a diagnostic, investigative, or therapeutic hypothesis. A new piece of information is then introduced, and the test-taker must judge, using a Likert-type scale, how this new data affects the likelihood or relevance of the hypothesis. Responses are scored by comparing the learner’s choices to those of a panel of experienced clinicians, with partial credit awarded based on the distribution of expert answers rather than a single correct response. This scoring system captures the variability in expert judgment and allows the SCT to measure concordance with expert reasoning rather than factual recall. Validity evidence for the SCT includes its ability to discriminate between levels of expertise, its positive impact on cognitive engagement, and its reliability when a sufficient number of items and panel members are used [3-5]. As such, SCTs are now used internationally in both undergraduate and postgraduate medical education, particularly in specialties that demand complex clinical judgment.

Artificial intelligence (AI) algorithms, such as large language models (LLMs), have developed in recent years, presenting a possible paradigm change in this field. Having been trained on extensive text datasets, LLMs are deep learning models that can produce language that is similar to that of a person, summarize intricate concepts, and even mimic domain-specific reasoning [6]. These models, as opposed to rule-based systems, generate context-aware responses, often with good fluency, by identifying patterns in unstructured data. LLMs have demonstrated the ability to replicate patient or clinician discourse, produce high-quality feedback, and perform at or near the passing threshold on United States Medical Licensing Examination–style questions [7-9].

In the field of LLMs used to generate SCTs, several studies have demonstrated the usefulness of this tool for creating clinical vignettes in medical education and other health sciences fields [10,11]. A recent study highlighted the educational quality of script concordance vignettes created using ChatGPT (OpenAI [11]). To use tools based on generative AI in the design of educational materials, and to improve efficiency while optimizing human and material resources, it is essential to evaluate the level of difficulty of the proposed scenarios and questions [12]. Moreover, to our knowledge, no study has yet demonstrated the ability of generative AI to embody the role of a clinical expert in selecting the most appropriate response to a given scenario and in providing feedback to learners.

The main objective of this project is to explore the performance of generative AI in its ability to create SCTs for undergraduate medical students and to embody the role of an expert. A secondary objective is to evaluate how medical learners perceive the difficulty level of the AI-generated SCTs, as well as their level of appreciation for each type of feedback provided.

## Methods

### Overview

**Figure 1.**
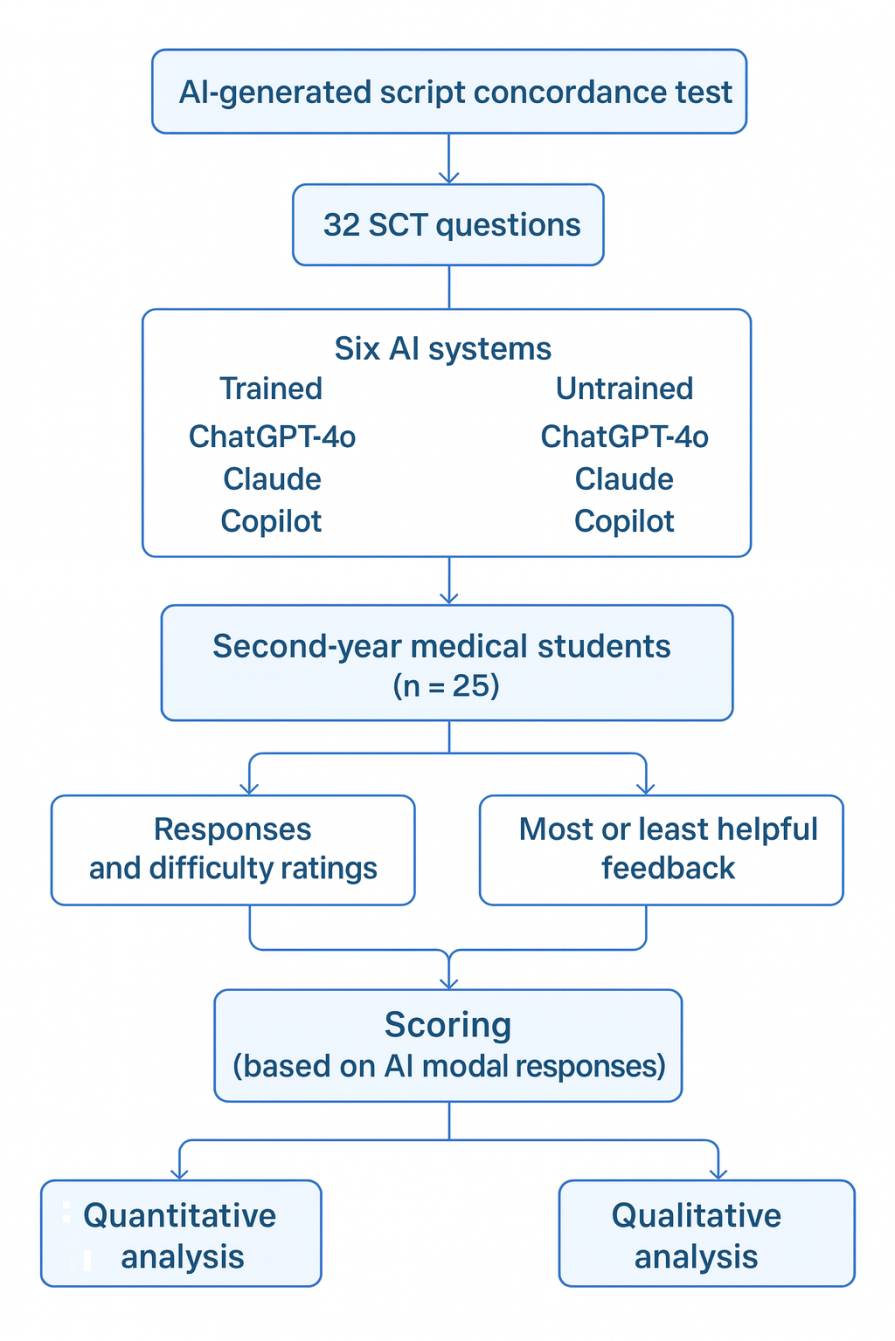
Protocol flow diagram for this study. AI: artificial intelligence; SCT: script concordance test.

This study used a cross-sectional, mixed methods experimental design to evaluate the performance of medical students on a generative AI–authored formative SCT and to assess the use of AI systems as content experts for both scoring and providing formative feedback. The flow diagram of this study is provided in Figure 1.

### Participants and Recruitment

The study was conducted in November 2024 at the Université de Montréal’s Faculty of Medicine. To be included, participants needed to be enrolled in the second year of the undergraduate medical education program, have completed their cardiology course, and be preparing for their respirology exam, having completed the related course materials. This timing allowed for the inclusion of clinical reasoning questions involving symptoms and signs that may relate to both systems, given their close physiological and clinical connections. An email was sent to all potential participants to register for this formative examination.

### Test Development and Structure

The SCT was developed using generative AI (ChatGPT-4o) following established guidelines for script concordance testing [13]. Four clinical vignettes (chronic obstructive pulmonary disease [COPD] exacerbation, hemoptysis, chest pain, and acute cough) were created, each containing 8 items for a total of 32. Prompts were standardized to ensure consistent vignette structure, 5-point Likert scaling (ranging from not likely to very likely, indicating how the new information would influence the hypothesis), and reasoning alignment across cardiopulmonary domains. Each item followed the classic SCT structure: (1) a clinical scenario introducing uncertainty, (2) a diagnostic, investigative, or therapeutic hypothesis, and (3) a new clinical finding. Items were designed and reviewed to reflect varying degrees of diagnostic ambiguity, probabilistic reasoning, and real-world uncertainty.

The full test, in French and its English translation, is provided in Multimedia Appendix 1. The following prompts were used to design the SCTs: (1) act as an expert in health sciences education at the university level, specializing in cardiology and pulmonology; (2) act as an expert in health sciences education at the university level, specializing in cardiology and pulmonology; (3) act as an expert in designing SCT vignettes; (4) generate a script concordance vignette that includes 8 questions based on a theme integrating both pulmonology and cardiology; and (5) create questions related to the vignette that begin with: “If you are considering ‘a diagnostic hypothesis’ and you find ‘a sign or symptom,’ how does this finding affect the likelihood of the hypothesis?” (using a Likert scale from −2 to +2 which refers to not likely to very likely).

While the AI-generated items were designed to follow the canonical SCT format (clinical vignette, hypothesis, new information, and Likert-scale inference), some questions did not fully adhere to this structure. This occurred when the generative model incorporated redundant information from the vignette into subsequent items, occasionally blurring the distinction between context and new data. The prompt did not explicitly constrain the model to exclude previously stated information, which likely contributed to partial format deviations.

### Use of Generative AI as an Expert Panel

The reference panel comprised 6 LLMs, chosen to balance diversity of reasoning with practical feasibility. Although the classical SCT format recommends 15‐20 human experts to ensure adequate response variability [5], LLMs can rapidly generate consistent probabilistic judgments across multiple iterations. Pilot testing indicated that 6 distinct model architectures provided sufficient variability in reasoning patterns to construct an aggregated scoring key while keeping the process computationally manageable for formative evaluation. These models were divided into two groups:

1. Trained AI experts (n=3): these systems (ChatGPT-4o, Claude 3.7 [Anthropic], and Microsoft 365 Copilot) were not fine-tuned or modified at the model-parameter level. Instead, each was contextually conditioned using course-specific documents (lecture notes and clinical guidelines from the cardiopulmonary block) that were embedded directly into the prompt context before generating responses. This approach (often referred to as in-context learning or prompt-based domain adaptation) allowed the models to access relevant curricular information without altering their underlying architecture.
2. Untrained AI experts (n=3): the same base models were used without additional curricular material or contextual embedding, representing their general-purpose configuration.

Each AI system was prompted to respond to all 32 SCT items as if it were a senior clinical expert. Their responses were used to establish scoring keys and to generate formative feedback explanations for each item. The modal response across all 6 AI experts served as the reference answer for scoring.

The prompts used to generate feedback were the same across the different AI systems. They were: (1) act as a medical expert in cardiology and pneumology; (2) act as a SCT expert; (3) for this vignette and each of its related questions ([Insert Vignette] and [Insert Questions]), please pick the most appropriate answer from the Likert scale; and (4) for each of your answers, please provide an explanation as if you were trying to explain your reasoning to a medical student.

### Student Completion and Data Collection

Participants completed a 32-question AI-generated SCT via a Google Form. For each item, students (1) selected a Likert-scale response, (2) rated the item’s perceived difficulty on a 7-point scale, (3) identified the most and least helpful feedback explanations among 6 anonymous AI-generated rationales, and (4) indicated whether they believed the question was AI- or human-authored. Open-ended comment boxes captured qualitative impressions. Completion was voluntary and anonymous, with implied consent through submission. The total estimated completion time was 35‐45 minutes. Responses were exported and cleaned in Microsoft Excel for analysis.

### Scoring and Analysis

Student answers were scored using the aggregate partial credit method: (1) one point was awarded if the student’s response matched the modal expert answer, (2) partial credit (eg, 0.75 and 0.5) was granted if the response matched a minority AI expert choice, and (3) zero points were given if the response did not match any AI panel member.

Descriptive statistics were computed for total scores, individual item performance, and perceived difficulty. Internal consistency was measured using Cronbach α. AI-to-AI and AI-to-student concordance was evaluated using Spearman rank correlation (ρ).

In the feedback evaluation component, student preferences for expert rationales were tabulated across all items. Each expert (AI system) received a score reflecting how frequently its feedback was selected as the “most helpful” and “least helpful,” allowing comparison between trained and untrained AI systems as feedback providers.

### Qualitative Analysis

Open-ended responses were analyzed using an inductive thematic approach. Comments were reviewed, coded iteratively, and grouped into major and minor themes by 2 independent reviewers. Discrepancies were resolved through discussion and consensus. Themes focused on student perceptions of the SCT format, feedback quality, and suggestions for improvement.

### Ethical Considerations

This study was approved by the Université de Montréal’s Research Ethics Committee in Education and Psychology under the project name 2024‐6168. Participation was anonymous and voluntary, with implied consent obtained through survey completion.

## Results

### Participants

A total of 25 students volunteered to complete the SCT in a formative, nonevaluative setting. Each student also evaluated the difficulty of each question, selected the most and least helpful expert feedback, and provided qualitative comments on individual questions and on the overall examination. Six generative AI systems (3 trained on course content and 3 untrained) completed the test and provided justifications for each item, which served as immediate feedback for the students.

### Student Performance

Among the 25 second-year medical students who completed the SCT, the average score was 22.8 out of 32 (SD 1.6), with individual scores ranging from 19.75 to 26.75. The distribution of scores approximated a normal curve with a slight left skew, indicating generally strong alignment between student responses and the AI expert panel, while still highlighting variability in performance. This range suggests that the AI-generated SCT was capable of differentiating clinical reasoning abilities among participants, supporting its discriminatory power even in a formative context. The full distribution of results for each question is provided in Table 1.

**Table 1.** Distribution of the test results (N=25).

Question (English)	Mean (SD)	Range (min-max)	Median (IQR)
Q1: To assess an acute COPD^a^ exacerbation, determine the impact of an oxygen saturation of 85%.	0.72 (0.15)	0.25-1	0.75 (0.75-0.75)
Q2: To evaluate acute pulmonary edema, judge the impact of a recent respiratory infection.	0.65 (0.2)	0-0.75	0.75 (0.75-0.75)
Q3: To assess the hypothesis of pulmonary embolism, determine the impact of dyspnea and tachycardia.	0.76 (0.17)	0.25-1	0.75 (0.75-0.75)
Q4: To evaluate myocardial dysfunction, judge the influence of a high heart rate.	0.56 (0.27)	0-1	0.75 (0.50-0.75)
Q5: To differentiate pulmonary edema from asthma exacerbation, assess the importance of a chest X-ray.	0.89 (0.21)	0.5-1	1 (1-1)
Q6: To evaluate a COPD exacerbation, determine how a history of smoking affects the hypothesis.	0.82 (0.15)	0.5-1	0.75 (0.75-1)
Q7: To guide diagnosis in respiratory distress, assess the importance of history-taking.	0.97 (0.11)	0.5-1	1 (1-1)
Q8: To diagnose pulmonary edema, judge the influence of acute electrocardiogram ischemia.	0.79 (0.19)	0.25-1	0.75 (0.75-1)
Q9: To assess tuberculosis as a differential diagnosis, evaluate the impact of night sweats and weight loss.	0.78 (0.08)	0.75-1	0.75 (0.75-0.75)
Q10: To assess the likelihood of chronic pulmonary hemoptysis, judge the influence of a history of bronchiectasis.	0.76 (0.13)	0.25-1	0.75 (0.75-0.75)
Q11: To assess life-threatening hemoptysis requiring urgent care, evaluate the impact of an oxygen saturation of 92%.	0.59 (0.32)	0-1	0.75 (0.25-0.75)
Q12: To evaluate bronchopulmonary cancer, determine the influence of a 30 pack-year smoking history.	0.86 (0.13)	0.75-1	0.75 (0.75-1)
Q13: To orient toward an infectious etiology like tuberculosis or pneumonia, assess the usefulness of the patient’s history.	1 (0)	1-1	1 (1-1)
Q14: To distinguish possible causes of hemoptysis, assess the importance of performing a chest X-ray.	1 (0)	1-1	1 (1-1)
Q15: To assess active bronchiectasis, judge the interaction between smoking history and risk of complications.	0.82 (0.14)	0.5-1	0.75 (0.75-1)
Q16: To diagnose tuberculosis-related hemoptysis, evaluate the influence of prior tuberculosis exposure.	0.86 (0.13)	0.75-1	0.75 (0.75-1)
Q17: To assess acute coronary syndrome (ACS), evaluate the impact of ST elevation in DII, DIII, and aVF^b^.	0.99 (0.05)	0.75-1	1 (1-1)
Q18: To evaluate atypical ACS presentation, assess the influence of diabetes.	0.75 (0.16)	0.5-1	0.75 (0.75-0.75)
Q19: To assess aortic dissection as a cause of pain, evaluate the impact of hypertension.	0.77 (0.1)	0.5-1	0.75 (0.75-0.75)
Q20: To evaluate suspected pneumothorax, judge how pain radiation influences the hypothesis.	0.29 (0.27)	0-1	0.25 (0.25-0.25)
Q21: To differentiate between ACS and pulmonary embolism, assess the importance of the electrocardiogram.	0.96 (0.12)	0.5-1	1 (1-1)
Q22: To evaluate pulmonary embolism, determine the influence of elevated blood pressure on the hypothesis.	0.55 (0.23)	0.25-0.75	0.75 (0.25-0.75)
Q23: To assess ACS likelihood, judge how chest pain radiation differs between male and female patients.	0.77 (0.2)	0.25-1	0.75 (0.75-1)
Q24: To determine the urgency of management, assess the influence of diabetes and hypertension.	0.78 (0.21)	0.5-1	0.75 (0.50-1)
Q25: To assess community-acquired pneumonia, evaluate the impact of initial fever on the hypothesis.	0.86 (0.13)	0.75-1	0.75 (0.75-1)
Q26: To evaluate treatment-induced cough, determine the influence of ACE inhibitor use.	0.72 (0.18)	0.25-1	0.75 (0.75-0.75)
Q27: To assess severe pneumonia requiring hospitalization, evaluate the impact of a normal oxygen saturation (97%).	0.15 (0.16)	0-0.5	0.25 (0-0.25)
Q28: To assess acute bronchitis, evaluate the impact of crackles on the hypothesis.	0.43 (0.28)	0-1	0.25 (0.25-0.75)
Q29: To distinguish between ACE-inhibitor-induced^c^ cough and acute respiratory infection, assess the usefulness of the history.	1 (0)	1-1	1 (1-1)
Q30: To assess upper respiratory tract infection, evaluate the influence of a 10-day duration of cough.	0.7 (0.2)	0.25-1	0.75 (0.75-0.75)
Q31: To differentiate pneumonia from bronchitis, assess the importance of a chest X-ray.	1 (0)	1-1	1 (1-1)
Q32: To guide the need for antibiotic treatment in suspected pneumonia, evaluate the role of initial fever and productive cough.	0.84 (0.2)	0.25-1	1 (0.75-1)

a COPD: chronic obstructive pulmonary disease.

b aVF: augmented vector foot.

c ACE: angiotensin-converting enzyme.

A more detailed item-level analysis revealed that questions with clearer clinical cues, such as Q1 (which evaluated the impact of hypoxemia [peripheral oxygen saturation of 85%] on the hypothesis of COPD exacerbation) and Q8 (which examined the influence of electrocardiogram findings on the diagnosis of acute pulmonary edema), had high rates of agreement with the expert panel. In these items, over 80% of student responses matched the most common expert answer, suggesting that students were comfortable applying well-established diagnostic patterns. In contrast, questions such as Q2 (regarding the influence of a recent respiratory infection on the hypothesis of pulmonary edema) and Q4 (assessing the relevance of tachycardia for myocardial dysfunction) showed greater variability in student responses, with less than 50% aligning with the modal expert choice. These items appeared to introduce more clinical ambiguity or demanded a higher level of inference, which may explain the broader dispersion of answers. Preliminary psychometric analysis demonstrated a Cronbach α of 0.76, indicating good internal consistency across the 32 items.

About 40% of students scored 25 or above, frequently aligning with expert responses even on more ambiguous questions. These students tended to report lower perceived difficulty and used the “slightly confirmed” response category more judiciously. The remaining 60% scored between 19 and 24 points and were more likely to select “non influenced” or “slightly confirmed” options, reflecting a more cautious reasoning style or uncertainty in applying new clinical information. Overall, the SCT demonstrated its utility in capturing variations in reasoning patterns and levels of diagnostic confidence among early clinical learners.

### Agreement Between AI and Medical Students

To evaluate the extent to which AI experts simulated expert reasoning, we examined concordance (the degree to which AI and student responses followed similar reasoning patterns) using Spearman rank correlation coefficient (ρ). This nonparametric measure assesses the strength and direction of association between 2 ranked sets of scores, where values closer to +1 indicate strong agreement and those near 0 indicate weak or no relationship.

Among the trained AI systems, ChatGPT-4o achieved the highest concordance with student responses (ρ=0.68; *P*<.001), followed by Claude (ρ=0.64) and Microsoft Copilot (ρ=0.61). These coefficients represent moderate-to-strong positive correlations, suggesting that trained models reasoned in ways closely aligned with students’ decision patterns. In contrast, untrained models showed weaker correlations (average ρ=0.41), reflecting less consistent or more generic reasoning.

Educationally, this implies that contextualized AI models can mirror how medical students weigh diagnostic information when course-specific data are embedded in their prompts. Such alignment supports the potential use of trained AI not only as an assessment proxy but also as a feedback tool capable of modeling clinically coherent reasoning.

The untrained AI models demonstrated lower levels of agreement. The untrained version of ChatGPT-4o showed a moderate correlation (ρ=0.48; *P*=.04), while Claude and Copilot, when untrained, showed weaker and statistically nonsignificant correlations of 0.42 (*P*=.06) and 0.34 (*P*=.08), respectively. These models tended to exhibit less consistent patterns, often defaulting to neutral or noncommittal answers such as “non influenced,” especially in items requiring nuanced interpretation of clinical signs. Their reasoning lacked the contextual anchoring present in the trained models and showed more variability across similar clinical scenarios. On average, the untrained models produced a correlation coefficient of 0.41, substantially lower than their trained counterparts.

### Perceived Difficulty

In addition to completing the 32 SCT items, students were asked to rate the perceived difficulty of each question on a 7-point Likert scale, ranging from 1 (“very easy”) to 7 (“very difficult”). This measure provided insight into how learners experienced the complexity and cognitive demands of the AI-generated test items. The mean perceived difficulty across all questions was 3.7, suggesting that students generally found the test to be of moderate difficulty. This aligns with the formative purpose of the SCT and indicates that the AI-generated items were accessible yet still challenging enough to stimulate clinical reasoning.

When analyzed at the item level, some questions emerged as consistently more difficult. In particular, Q2 (which assessed how a recent respiratory infection influences the hypothesis of acute pulmonary edema) and Q4 (which explored the relevance of tachycardia in the context of suspected myocardial dysfunction) received higher average difficulty ratings, with mean scores exceeding 4.5 (SD 0.9). These questions involved ambiguous or indirect relationships between the new information and the hypothesis, requiring students to reason within clinical gray zones where data interpretation is not straightforward. In contrast, items that presented clear diagnostic anchors, such as Q1 (hypoxemia in COPD) and Q8 (electrocardiogram ischemia in pulmonary edema), were rated as significantly easier, with average difficulty scores below 3.0. These findings suggest that item clarity and familiarity with pathophysiological mechanisms strongly influence perceived complexity.

Furthermore, there was no statistically significant difference in perceived difficulty between items believed by students to be human-authored and those they believed to be AI-authored (*t* test; *P*=.47). As all items were in fact AI-generated, this finding reflects students’ perceived authenticity of the AI-written vignettes rather than a comparison with true human-authored items. This finding supports the face validity of AI-generated items, indicating that they are perceived as authentic and comparable in challenge to traditional, faculty-written SCT items. Several students even remarked in open comments that they were “unable to tell” which questions had been generated by AI, further supporting the indistinguishability of AI-authored content when properly crafted.

Cluster analysis of difficulty ratings also revealed subtle differences in student subgroups. High-performing students (as defined by SCT scores ≥25/32) tended to rate the test as less difficult overall (mean 3.3, SD 0.8) compared to their peers (mean 3.9, SD 0.8), which may reflect their greater familiarity with clinical reasoning or more efficient script activation. In addition, free-text comments frequently noted that clinical ambiguity (rather than item length or language) was the primary factor contributing to perceived difficulty. Students consistently emphasized that the challenge came from “figuring out what matters most” in the presence of partial or conflicting clinical cues. The difficulty ratings offer an important validation metric for AI-generated SCTs. The overall moderate difficulty, the range of item-specific variation, and the lack of perceived discrepancy between AI- and human-generated content all point toward a well-calibrated test.

Spearman correlation analysis between item difficulty ratings and alignment with trained AI responses yielded a modest positive trend (ρ=0.32; *P*=.08), suggesting that items more closely matching trained AI reasoning were often perceived as less difficult. This pattern hints that when AI-generated reasoning aligns with instructional scripts, students experience smoother cognitive processing and reinforce the pedagogical relevance of model calibration.

### Evaluation of AI-Expert Feedback

In addition to rating the difficulty of each SCT item, students were asked to select the expert feedback they considered most helpful and least helpful among the options provided. For every SCT question, 6 expert rationales were made available to the medical students by the AI systems (trained and untrained). Students were instructed to base their selection on 3 specific criteria: clarity, clinical relevance, and overall educational value. This evaluation provided insight not only into the perceived credibility of AI-generated explanations but also into the comparative strengths of different expert types in formative feedback contexts.

Across all 32 questions, AI-generated feedback (especially from models trained on the course material) was selected as helpful in 62.5% of instances. Among these, ChatGPT-4o (trained) was most frequently chosen, followed by Claude (trained) and Copilot (trained). Students frequently cited the clarity of language, conciseness, and alignment with taught clinical reasoning strategies as reasons for preferring these explanations. Trained AI feedback was especially appreciated in items requiring pathophysiological reasoning, such as those related to acute coronary syndromes and differential diagnoses in respiratory presentations. The consistent use of structured logic and evidence-based phrasing contributed to a perception of trustworthiness and educational quality.

In contrast, the feedback generated by untrained AI systems was selected as most helpful in only 9.4% of instances and more frequently identified as least useful. The untrained models often produced generic or excessively cautious explanations, relying on vague phrases such as “may support the hypothesis” or “needs further investigation” without directly engaging with the specific clinical cues provided in the vignette. These models occasionally failed to recognize important pathophysiological links, leading to misalignment with students’ expectations. Several students noted that while the untrained AI responses were not necessarily incorrect, they lacked the pedagogical precision needed for effective feedback.

### Qualitative Feedback From Students

#### Overview

To complement the quantitative analyses, students were invited to provide open-ended comments after each SCT question and at the end of the test. These narrative responses were analyzed using an inductive thematic analysis. A total of 94 discrete comments were submitted by the 25 participating students. From these, three major themes were identified: (1) ambiguity and interpretation of Likert-scale options, (2) educational value of SCTs, and (3) recommendations for improvement in item design and feedback delivery. Key themes and examples of quotes are provided in Table 2.

**Table 2.** Key themes, descriptions, and their representative quotes.

Theme	Description	Representative quotes
Ambiguity and interpretation of Likert-scale options	Students expressed uncertainty distinguishing between closely-related options (”slightly confirmed“ vs ”confirmed”), reflecting the inherent challenge of probabilistic reasoning under limited data.	“It’s really hard to know when to say ‘confirmed’ vs ‘slightly confirmed’ without lab results.” “Sometimes the answer depends on how I imagine the rest of the case.”
Educational value of the SCT^a^ format	Learners appreciated how SCTs stimulated reflective reasoning and moved beyond right-wrong logic, reinforcing their understanding of uncertainty.	“This test made me think like a real clinician.” “I liked how the questions made me weigh the value of each piece of information.”
Recommendations for improvement	Students suggested adding contextual information (eg, vital signs and comorbidities) and calibration examples to improve clarity and confidence.	“More case details would help me justify my choice better.” “Before the real test, I would have liked an example with expert explanation.”

a SCT: script concordance test.

#### Ambiguity and Interpretation of Likert-Scale Options

A recurrent theme in the comments related to students’ uncertainty in differentiating between closely related answer choices. Many expressed difficulties in deciding whether a new piece of clinical information “slightly confirmed” versus “confirmed” a hypothesis, particularly when the vignette lacked strong diagnostic anchors.

This theme reflects a broader cognitive tension between probabilistic reasoning and binary decision-making in early clinical training. Students seemed to grasp the script concordance philosophy but struggled with the semantic granularity required by the scale, particularly in low-certainty situations.

#### Educational Value of the SCT Format

Despite difficulties with Likert scales, students generally expressed strong appreciation for the SCT format as a learning tool. Many found it stimulated reflection on clinical uncertainty and offered a welcome departure from the right/wrong dichotomy typical of multiple-choice assessments.

Students also praised the opportunity to compare their reasoning to experts, particularly when feedback was provided in structured, explanatory formats. Some noted that this type of test encouraged them to revisit and revise their clinical scripts, reinforcing clinical reasoning pathways rather than isolated facts.

#### Recommendations for Improvement

Several students offered constructive suggestions aimed at improving the test format and clarity. One prominent comment was the request for more context in the vignettes, such as vital signs, lab values, or imaging findings. Students felt that additional details would allow them to apply reasoning with greater precision and confidence.

Another frequently mentioned point was the desire for guidance or training on how to use the Likert scale. Some students suggested including sample questions or expert rationales before the actual test to calibrate their expectations.

Interestingly, several students highlighted their surprise at the quality of feedback provided by AI systems. These comments were often framed with curiosity or mild skepticism, indicating an openness to AI as a pedagogical tool, contingent on quality and relevance.

While students appreciated the reflective and authentic nature of the format, they also identified areas where clearer structure or training could enhance their experience. Their willingness to accept AI-generated feedback, when well-crafted, suggests that such technologies could play a productive role in clinical education, particularly when embedded within thoughtful educational frameworks. This thematic analysis suggests that future iterations of the SCT should include providing students with explicit guidance and calibration examples on how to interpret the Likert scale; enhancing vignettes with additional contextual cues (eg, vital signs or comorbidities) to support more confident reasoning; incorporating trained AI models as feedback generators, with ensuring faculty review for quality assurance; and designing preparatory activities that help learners understand how to engage with probabilistic reasoning in uncertain clinical scenarios.

## Discussion

### Principal Findings

This study explores the performance of generative AI in its ability to create SCTs for undergraduate medical students and to embody the role of an expert model by fully designing, scoring, and providing feedback for an SCT in undergraduate medical education. Among the 25 second-year medical students who completed the AI-generated SCT, the mean score was 22.8 out of 32, with a distribution that reflected meaningful variation in clinical reasoning patterns. Students generally aligned well with expert-modeled responses, particularly on items with strong pathophysiological anchors, while questions requiring greater inferential reasoning yielded more dispersed answers. Trained AI systems (those provided with course-specific materials) achieved the highest concordance with both student performance and expected reasoning pathways, offering feedback that was frequently rated as the most helpful. Untrained AI models, in contrast, were perceived as less pedagogically effective and exhibited weaker correlations with student responses. The SCT also performed reliably, with a Cronbach α of 0.76, and was rated by students as moderately difficult, further reinforcing the test’s capacity to capture meaningful distinctions in clinical reasoning. Interestingly, preliminary trends suggested that students with higher SCT scores tended to select feedback from trained AI systems more often, implying that the pedagogical structure of these explanations may resonate most with learners who already demonstrate stronger reasoning frameworks. This observation supports the idea that trained AI feedback could help reinforce expert-like reasoning scripts, though confirmatory analyses in larger samples are needed.

### Comparison With Previous Work

The integration of AI in formative clinical assessment tools, such as SCTs, is still at an early stage, but this study offers an early applied example of how LLMs can simulate aspects of expert reasoning within a script-concordance framework when appropriately contextualized. While previous research has already demonstrated the clinical reasoning capabilities of LLMs in diverse testing environments (eg, Nori et al [14] and Singhal et al [6]), this work extends these findings to a formative educational setting by integrating AI-generated items, scoring, and feedback within a single workflow. While previous studies have explored LLMs’ ability to answer multiple-choice questions or United States Medical Licensing Examination–style questions, those formats tend to rely on recall and fixed answers [15,16]. In contrast, SCTs demand probabilistic thinking and tolerance of ambiguity, qualities that better reflect real-world medical clinical reasoning and thus could be used in exams to test clinical reasoning. The ability of trained LLMs in this study to mirror the distribution of expert responses (and to provide feedback that students found clear and pedagogically relevant) suggests a step forward in the use of AI not merely as a content generator, but as a cognitive proxy for experienced clinicians.

This study also aligns with findings from recent work examining AI’s capacity to replicate reflective thinking patterns. For instance, Nori et al [14] highlighted that LLMs such as GPT-4 are increasingly capable of “meta-cognitive” behavior, adapting their justifications based on context and complexity. The findings support this, especially in items where the trained AI systems offered nuanced rationales that resonated with learners. Furthermore, the indistinguishability between human- and AI-authored feedback, as perceived by students in this study, aligns with previous reports by Lee and Song [17], who found that students often could not discern between AI-generated and expert-written content when both were of high quality. Importantly, students in this cohort demonstrated openness to AI-generated feedback, provided it was contextually accurate and clearly structured, suggesting that AI can be accepted as a trusted educational voice when properly curated.

Nonetheless, this project also illustrates the current limitations of general-purpose LLMs. Untrained models, though capable of generating grammatically correct responses, frequently defaulted to cautious or generic reasoning, especially in questions requiring interpretation of subtle clinical cues. This reduced their pedagogical value and undermined student trust. These findings are consistent with recent critiques from Arora and Arora [18], who argue that untrained LLMs lack the contextual grounding needed for expert-level interpretation, particularly in nuanced or culturally specific clinical scenarios. As such, effective integration of AI into medical education likely hinges on customized fine-tuning, prompt engineering, and ongoing human oversight to ensure that AI explanations remain accurate, relevant, and educationally meaningful.

### Limitations

This study has several limitations. The sample size was modest and drawn from a single institution, which may limit generalizability. The test was also formative in nature, and students may not have engaged with the same level of cognitive effort as they would in summative settings. It is also possible that only students who expressed an interest in AI were accepted to participate in this SCT, which may affect the generalizability of the qualitative components of this study. In addition, while the AI models were trained on course materials, their responses still depended on prompt clarity and token limits, which may have shaped their output in subtle ways. No direct comparison was made between AI and human expert panels on the same test, which restricts our ability to determine whether the observed concordance between AI and student reasoning reflects genuine expert-level clinical judgment. As such, these findings demonstrate internal validity and feasibility rather than equivalence to clinician consensus. Future studies should include parallel human-expert panels to establish criterion validity and to calibrate the pedagogical quality of AI-generated scoring keys and feedback. As this was a single-round implementation, long-term effects on clinical reasoning or retention were not assessed. Future studies should explore how repeated exposure to AI-generated SCTs impacts learning trajectories and whether hybrid models (AI and human feedback) offer added value over AI alone.

Finally, a further limitation relates to the potential for hallucinations or inaccurate information in AI-generated explanations. While all outputs were reviewed for face plausibility and internal consistency, no formal content validation against gold-standard references was performed. As highlighted by Masters [19], generative AI systems can produce fabricated or misleading references and medical details, a phenomenon known as “AI hallucination.” This risk underscores the importance of treating AI-generated feedback as supplementary rather than authoritative, especially in formative educational contexts. Future implementations should incorporate systematic expert review of AI-generated feedback to ensure clinical accuracy and safeguard learners from misinformation.

### Conclusions

As AI technologies evolve and become increasingly embedded in medical education, this study offers novel insight into how LLMs can assume the role traditionally occupied by human experts in clinical reasoning assessments. By designing and scoring an SCT entirely with generative AI systems, and by leveraging both trained and untrained models to simulate expert feedback, we demonstrated that AI can produce assessment content that is not only functionally valid but also well-received by learners. The high degree of alignment between student responses and trained AI-generated reference panels, alongside the positive reception of AI-authored feedback, suggests that these technologies hold real promise for supporting formative assessment. While distinctions remain between the effectiveness of trained versus untrained models, this study illustrates that with proper calibration, AI systems can enhance both the efficiency and pedagogical depth of clinical education. These findings open the door for further research into scalable, AI-assisted assessment models that can flexibly support reasoning under uncertainty, which is an essential competency in modern medical practice. Beyond feasibility, this work underscores the transformative potential of generative AI to democratize access to high-quality formative assessment. In academic environments where expert availability and time are limited, AI-assisted SCT generation could substantially reduce faculty workload, expand curricular coverage, and enable rapid adaptation of tests to evolving learning objectives. By lowering the resource barrier traditionally associated with expert panel-based assessment, this approach positions AI as a potential catalyst for sustainable, scalable innovation in medical education globally.

## Supplementary material

10.2196/76618 Multimedia Appendix 1 Script concordance test (French and English).

## Abbreviations

AI: artificial intelligence
COPD: chronic obstructive pulmonary disease
LLM: large language model
SCT: script concordance test
