# Supplementary material for: Teaching Clinical Reasoning in Health Care Professions Learners Using AI-Generated Script Concordance Tests: Mixed Methods Formative Evaluation
Source: JMIR Form Res. 2025 Nov 20;9:e76618. doi: 10.2196/76618 (PMC12634011; doi:10.2196/76618)
Supplement: Multimedia Appendix 1 [file formative-v9-e76618-s001.docx]

## Examen formatif

### Dyspnée

**Scénario clinique :**

Un homme de 67 ans se présente aux urgences avec une dyspnée aiguë survenue il y a deux heures. Il décrit une sensation d’oppression thoracique et une difficulté progressive à respirer. Il est diaphorétique et légèrement confus. Sa fréquence respiratoire est de 28/min, sa saturation en oxygène est de 85 % en air ambiant, sa pression artérielle est de 150/90 mmHg et sa fréquence cardiaque est de 110 battements/min. Il a des antécédents d’hypertension, de tabagisme (20 paquets-années) et a eu une infection respiratoire il y a deux semaines. Il ne prend pas de traitement régulier pour une pathologie pulmonaire connue.

**Q1 :** Si vous pensez à une exacerbation aiguë de la maladie pulmonaire obstructive chronique (MPOC) dans ce cas, quel est l’impact de la saturation d’oxygène à 85 % ?

- Hypothèse confirmée
- Hypothèse légèrement confirmée
- Hypothèse non influencée
- Hypothèse légèrement infirmée
- Hypothèse infirmée

**Q2 :** Si vous pensez à un œdème aigu du poumon, comment l’antécédent d’infection respiratoire récente influence-t-il votre hypothèse ?

- Hypothèse confirmée
- Hypothèse légèrement confirmée
- Hypothèse non influencée
- Hypothèse légèrement infirmée
- Hypothèse infirmée

**Q3 :** Quel impact l’examen montre-t-il pour une embolie pulmonaire en raison de la dyspnée et de la tachycardie associée ?

- Hypothèse confirmée
- Hypothèse légèrement confirmée
- Hypothèse non influencée
- Hypothèse légèrement infirmée
- Hypothèse infirmée

**Q4 :** Si vous envisagez une dysfonction myocardique comme diagnostic possible, comment la fréquence cardiaque élevée influence-t-elle cette hypothèse ?

- Hypothèse confirmée
- Hypothèse légèrement confirmée
- Hypothèse non influencée
- Hypothèse légèrement infirmée
- Hypothèse infirmée

**Q5 :** Quelle serait l’importance d’une radiographie pulmonaire pour différencier un œdème pulmonaire d’une exacerbation de l’asthme ?

- Très importante
- Importante
- Modérément importante
- Peu importante
- Non importante

**Q6 :** L'antécédent de tabagisme modifie-t-il l'hypothèse d'une exacerbation de la MPOC ?

- Hypothèse confirmée
- Hypothèse légèrement confirmée
- Hypothèse non influencée
- Hypothèse légèrement infirmée
- Hypothèse infirmée

**Q7 :** En présence de signes de détresse respiratoire, dans quelle mesure l’anamnèse est-elle essentielle pour orienter le diagnostic ?

- Essentielle
- Très utile
- Modérément utile
- Peu utile
- Inutile

**Q8 :** Comment une ischémie aiguë à l’ECG influencerait-elle votre diagnostic d’œdème aigu du poumon ?

- Hypothèse confirmée
- Hypothèse légèrement confirmée
- Hypothèse non influencée
- Hypothèse légèrement infirmée
- Hypothèse infirmée

### Hémoptysies

**Scénario clinique**

Une femme de 55 ans se présente aux urgences avec des épisodes de toux productive avec du sang depuis trois jours. Elle rapporte une perte de poids involontaire, de la fatigue et des sueurs nocturnes. Elle est fumeuse depuis 30 ans (30 paquets-années) et a des antécédents de bronchiectasies. À l'examen, elle est légèrement tachypnéique, sa saturation en oxygène est de 92 % en air ambiant, et sa pression artérielle est de 130/85 mmHg.

**Questions**

**Q1: Si vous envisagez la tuberculose comme diagnostic différentiel, quel est l’impact des sueurs nocturnes et de la perte de poids ?**

- Hypothèse confirmée
- Hypothèse légèrement confirmée
- Hypothèse non influencée
- Hypothèse légèrement infirmée
- Hypothèse infirmée

**Q2: Comment l’antécédent de bronchiectasies influence-t-il la probabilité d’une hémoptysie d'origine pulmonaire chronique ?**

- Hypothèse confirmée
- Hypothèse légèrement confirmée
- Hypothèse non influencée
- Hypothèse légèrement infirmée
- Hypothèse infirmée

**Q3: Quel est l’impact de la saturation en oxygène à 92 % sur la probabilité d’une hémoptysie massive nécessitant une prise en charge urgente ?**

- Hypothèse confirmée
- Hypothèse légèrement confirmée
- Hypothèse non influencée
- Hypothèse légèrement infirmée
- Hypothèse infirmée

**Q4: Comment la présence d'un tabagisme à 30 paquets-années influence-t-elle votre hypothèse d'un cancer bronchopulmonaire ?**

- Hypothèse confirmée
- Hypothèse légèrement confirmée
- Hypothèse non influencée
- Hypothèse légèrement infirmée
- Hypothèse infirmée

**Q5: Dans quelle mesure l’anamnèse de la patiente est-elle utile pour orienter vers une étiologie infectieuse comme la tuberculose ou la pneumonie ?**

- Essentielle
- Très utile
- Modérément utile
- Peu utile
- Inutile

**Q6: Dans le cas d’une hémoptysie, quelle est l'importance de réaliser une radiographie pulmonaire pour distinguer les diagnostics potentiels ?**

- Très importante
- Importante
- Modérément importante
- Peu importante
- Non importante

**Q7: Si vous pensez à une bronchiectasie active, dans quelle mesure les antécédents de tabagisme augmentent-ils le risque de complications ?**

- Hypothèse confirmée
- Hypothèse légèrement confirmée
- Hypothèse non influencée
- Hypothèse légèrement infirmée
- Hypothèse infirmée

**Q8: Comment une confirmation d’exposition antérieure à la tuberculose influencerait-elle le diagnostic d’une hémoptysie d’origine tuberculeuse ?**

- Hypothèse confirmée
- Hypothèse légèrement confirmée
- Hypothèse non influencée
- Hypothèse légèrement infirmée
- Hypothèse infirmée

### Douleurs thoraciques

**Scénario clinique**

Un homme de 58 ans se présente aux urgences pour une douleur thoracique intense, survenue de manière aiguë il y a 30 minutes. Il décrit la douleur comme un serrement irradiant vers le bras gauche et la mâchoire. Il est anxieux, diaphorétique, et légèrement tachycarde avec une pression artérielle de 160/95 mmHg. Il est connu pour hypertension et diabète de type 2. À l'ECG, on note un sus-décalage du segment ST en DII, DIII, et aVF.

**Questions**

1. Si vous suspectez un syndrome coronarien aigu (SCA) dans ce cas, quel est l’impact du sus-décalage ST en DII, DIII, et aVF ?
   - Hypothèse confirmée
   - Hypothèse légèrement confirmée
   - Hypothèse non influencée
   - Hypothèse légèrement infirmée
   - Hypothèse infirmée
2. Comment la présence de diabète influence-t-elle votre hypothèse d’un SCA avec présentation atypique ?
   - Hypothèse confirmée
   - Hypothèse légèrement confirmée
   - Hypothèse non influencée
   - Hypothèse légèrement infirmée
   - Hypothèse infirmée
3. Quel est l’impact de l’hypertension sur la probabilité d’une dissection aortique comme cause de la douleur ?
   - Hypothèse confirmée
   - Hypothèse légèrement confirmée
   - Hypothèse non influencée
   - Hypothèse légèrement infirmée
   - Hypothèse infirmée
4. Si vous suspectez un pneumothorax, comment l’irradiation de la douleur influence-t-elle cette hypothèse ?
   - Hypothèse confirmée
   - Hypothèse légèrement confirmée
   - Hypothèse non influencée
   - Hypothèse légèrement infirmée
   - Hypothèse infirmée
5. Quelle est l’importance de l’ECG dans la différenciation entre un SCA et une embolie pulmonaire ?
   - Très importante
   - Importante
   - Modérément importante
   - Peu importante
   - Non importante
6. Si vous pensez à une embolie pulmonaire, dans quelle mesure la pression artérielle élevée influence-t-elle cette hypothèse ?
   - Hypothèse confirmée
   - Hypothèse légèrement confirmée
   - Hypothèse non influencée
   - Hypothèse légèrement infirmée
   - Hypothèse infirmée
7. Comment la présentation de douleur thoracique avec irradiation chez ce patient masculin influence-t-elle la probabilité d'un SCA par rapport à un patient de sexe féminin ?
   - Hypothèse confirmée
   - Hypothèse légèrement confirmée
   - Hypothèse non influencée
   - Hypothèse légèrement infirmée
   - Hypothèse infirmée
8. Comment l'anamnèse de diabète et d’hypertension du patient influence-t-elle la nécessité d’une prise en charge urgente ?
   - Prise en charge urgente nécessaire
   - Prise en charge urgente légèrement influencée
   - Prise en charge non influencée
   - Prise en charge légèrement infirmée
   - Prise en charge infirmée

### Toux

**Scénario clinique**

Un homme de 45 ans consulte pour une toux persistante depuis 10 jours. Il mentionne que la toux est productive, surtout le matin, et qu'il a eu de la fièvre (38,5 °C) pendant les trois premiers jours. Il n’a pas d’antécédents respiratoires significatifs, mais il a récemment commencé un traitement par inhibiteurs de l'enzyme de conversion (IEC) pour son hypertension. À l'examen, sa saturation en oxygène est de 97 % en air ambiant, et on note quelques râles crépitants basaux bilatéraux.

**Q1: Si vous suspectez une pneumonie communautaire dans ce cas, quel est l’impact de la fièvre initiale sur cette hypothèse ?**

- Hypothèse confirmée
- Hypothèse légèrement confirmée
- Hypothèse non influencée
- Hypothèse légèrement infirmée
- Hypothèse infirmée

**Q2: Comment l’usage d’un IECA influence-t-il votre hypothèse d’une toux secondaire au traitement ?**

- Hypothèse confirmée
- Hypothèse légèrement confirmée
- Hypothèse non influencée
- Hypothèse légèrement infirmée
- Hypothèse infirmée

**Q3: Quel est l’impact de la saturation en oxygène normale (97 %) sur la probabilité d’une pneumonie sévère nécessitant une hospitalisation ?**

- Hypothèse confirmée
- Hypothèse légèrement confirmée
- Hypothèse non influencée
- Hypothèse légèrement infirmée
- Hypothèse infirmée

**Q4: Si vous envisagez une bronchite aiguë, quel est l’impact des râles crépitants sur cette hypothèse ?**

- Hypothèse confirmée
- Hypothèse légèrement confirmée
- Hypothèse non influencée
- Hypothèse légèrement infirmée
- Hypothèse infirmée

**Q5: Dans quelle mesure l’anamnèse est-elle utile pour distinguer une toux liée à un IECA d’une infection respiratoire aiguë ?**

- Essentielle
- Très utile
- Modérément utile
- Peu utile
- Inutile

**Q6: Comment la durée de 10 jours de la toux influence-t-elle votre hypothèse d'une infection des voies respiratoires supérieures ?**

- Hypothèse confirmée
- Hypothèse légèrement confirmée
- Hypothèse non influencée
- Hypothèse légèrement infirmée
- Hypothèse infirmée

**Q7: Quelle est l’importance d’une radiographie pulmonaire pour différencier une pneumonie d’une bronchite ?**

- Très importante
- Importante
- Modérément importante
- Peu importante
- Non importante

**Q8: Si vous suspectez une pneumonie, dans quelle mesure la fièvre initiale et la productivité de la toux orientent-elles vers la nécessité d’un traitement antibiotique ?**

- Traitement antibiotique fortement indiqué
- Traitement antibiotique légèrement indiqué
- Traitement antibiotique non influencé
- Traitement antibiotique légèrement contre-indiqué
- Traitement antibiotique contre-indiqué

## Exemple type de réponses des experts simulés (LLMs) :

**Q4 :** Si vous envisagez une dysfonction myocardique comme diagnostic possible, comment la fréquence cardiaque élevée influence-t-elle cette hypothèse ?

**Réponses des experts:**

**Expert 1 :Hypothèse confirmée:**La tachycardie peut être une réponse compensatoire dans une dysfonction myocardique, augmentant la probabilité d’insuffisance cardiaque.
**Expert 2 :Hypothèse légèrement confirmée** : Une fréquence cardiaque élevée peut indiquer une surcharge de travail pour le cœur, souvent observée dans la dysfonction myocardique. **Expert 3 :Hypothèse confirmée.** Une fréquence cardiaque élevée peut être un signe d'ischémie myocardique.   **Expert 4 :Hypothèse légèrement confirmée** : Une fréquence cardiaque élevée peut être un signe de dysfonction myocardique, en particulier en cas d’insuffisance cardiaque aiguë ou d’ischémie myocardique, qui peuvent entraîner une réponse tachycardique compensatoire. Associée à d'autres signes comme l'oppression thoracique et la dyspnée aiguë, cette tachycardie renforce légèrement l'hypothèse d'une dysfonction myocardique sans pour autant la confirmer complètement, car elle peut aussi être présente dans d’autres pathologies aiguës. **Expert 5 :Hypothèse légèrement confirmée:**  La fréquence cardiaque élevée peut être un signe de dysfonction myocardique, car le cœur peut battre plus rapidement pour compenser une diminution de la fonction de pompage. Cela peut indiquer une surcharge de travail pour le cœur, souvent observée dans les cas d’insuffisance cardiaque ou de dysfonction myocardique.
**Expert 6 :Hypothèse confirmée:**

1. La tachycardie (FC=110/min) s'inscrit dans un tableau clinique très évocateur :
   - Dyspnée aiguë
   - Oppression thoracique
   - Diaphorèse
   - Désaturation à 85%
2. Cette tachycardie est particulièrement significative car :
   - Elle représente un mécanisme compensatoire typique d'une défaillance cardiaque
   - Elle s'accompagne de symptômes classiques de dysfonction myocardique
   - Le patient présente des facteurs de risque cardiovasculaires (HTA, tabagisme)
3. Dans ce contexte clinique spécifique, la tachycardie :
   - Est un signe cardinal de dysfonction myocardique
   - Peut être à la fois cause et conséquence du tableau
   - S'intègre parfaitement dans le tableau d'une défaillance cardiaque aiguë
   - Cette association de signes, dont la tachycardie, est suffisamment caractéristique pour confirmer (et non pas seulement légèrement confirmer) l'hypothèse d'une dysfonction myocardique.

Selon-vous, quelle réponse d'expert est la plus utile?

Expert 1

Expert 2

Expert 3

Expert 4

Expert 5

Expert 6

Selon-vous, quelle réponse d'expert est la moins utile?

Expert 1

Expert 2

Expert 3

Expert 4

Expert 5

Expert 6

Cette question a-t-elle été rédigée par intelligence artificielle?

Oui

Non

Difficulté de la question

Très facile

1

2

3

4

5

6

7

8

9

10

Très difficile

Commentaires libres sur la question

**Dyspnea**

**Clinical Scenario:**

A 67-year-old man presents to the emergency department with acute dyspnea that started two hours ago. He describes a sensation of chest tightness and progressive difficulty breathing. He is diaphoretic and slightly confused. His respiratory rate is 28/min, oxygen saturation is 85% on room air, blood pressure is 150/90 mmHg, and heart rate is 110 bpm. He has a history of hypertension, smoking (20 pack-years), and had a respiratory infection two weeks ago. He does not take regular treatment for any known pulmonary condition.

**Questions:**

- **Q1:** If you consider an acute exacerbation of chronic obstructive pulmonary disease (COPD) in this case, what is the impact of the oxygen saturation at 85%?
- **Q2:** If you consider acute pulmonary edema, how does the recent respiratory infection influence your hypothesis?
- **Q3:** What is the impact of the physical examination findings on the hypothesis of pulmonary embolism, given the associated dyspnea and tachycardia?
- **Q4:** If you consider myocardial dysfunction as a possible diagnosis, how does the elevated heart rate influence your hypothesis?
- **Q5:** What is the importance of a chest X-ray in differentiating pulmonary edema from an asthma exacerbation?
- **Q6:** Does the smoking history modify the hypothesis of COPD exacerbation?
- **Q7:** In the presence of signs of respiratory distress, how essential is the history to guide diagnosis?
- **Q8:** How would acute ischemia on ECG influence your diagnosis of acute pulmonary edema?

Answer Choices (for Q1–Q4, Q6, Q8):

- Hypothesis confirmed
- Hypothesis slightly confirmed
- Hypothesis not influenced
- Hypothesis slightly refuted
- Hypothesis refuted

Answer Choices (for Q5, Q7):

- Very important / Essential
- Important / Very useful
- Moderately important / Moderately useful
- Slightly important / Slightly useful
- Not important / Useless

**Hemoptysis**

**Clinical Scenario:**

A 55-year-old woman presents to the emergency department with productive cough with blood for three days. She reports unintentional weight loss, fatigue, and night sweats. She is a smoker (30 pack-years) and has a history of bronchiectasis. On examination, she is slightly tachypneic, oxygen saturation is 92% on room air, and blood pressure is 130/85 mmHg.

**Questions:**

- **Q1:** If considering tuberculosis as a differential diagnosis, what is the impact of night sweats and weight loss?
- **Q2:** How does the history of bronchiectasis influence the probability of chronic pulmonary hemoptysis?
- **Q3:** What is the impact of 92% oxygen saturation on the likelihood of massive hemoptysis requiring urgent management?
- **Q4:** How does a 30 pack-year smoking history influence your hypothesis of bronchopulmonary cancer?
- **Q5:** How useful is the patient's history in orienting towards an infectious etiology like tuberculosis or pneumonia?
- **Q6:** In the case of hemoptysis, how important is a chest X-ray to differentiate potential diagnoses?
- **Q7:** If you suspect active bronchiectasis, how does the smoking history increase the risk of complications?
- **Q8:** How would confirmation of prior tuberculosis exposure influence the diagnosis of tuberculous hemoptysis?

Answer Choices same as Dyspnea section.

**Chest Pain**

**Clinical Scenario:**

A 58-year-old man presents to the emergency department with intense chest pain that started acutely 30 minutes ago. He describes the pain as squeezing, radiating to the left arm and jaw. He is anxious, diaphoretic, and slightly tachycardic, with blood pressure 160/95 mmHg. He has known hypertension and type 2 diabetes. ECG shows ST-segment elevation in leads II, III, and aVF.

**Questions:**

- **Q1:** If you suspect acute coronary syndrome (ACS) in this case, what is the impact of the ST-segment elevation in leads II, III, and aVF?
- **Q2:** How does the presence of diabetes influence your hypothesis of an ACS with atypical presentation?
- **Q3:** What is the impact of hypertension on the likelihood of an aortic dissection causing the pain?
- **Q4:** If you suspect a pneumothorax, how does the radiation of pain influence your hypothesis?
- **Q5:** What is the importance of ECG in differentiating between ACS and pulmonary embolism?
- **Q6:** If you consider pulmonary embolism, how does elevated blood pressure influence your hypothesis?
- **Q7:** How does the presentation of chest pain with radiation in this male patient influence the probability of ACS compared to a female patient?
- **Q8:** How does the patient's history of diabetes and hypertension influence the need for urgent management?

Answer Choices same as Dyspnea section, with Q8 answer choices adapted to:

- Urgent management required
- Urgent management slightly influenced
- Urgent management not influenced
- Urgent management slightly refuted
- Urgent management refuted

**Cough**

**Clinical Scenario:**

A 45-year-old man consults for a persistent cough lasting 10 days. He notes that the cough is productive, especially in the morning, and that he had a fever (38.5°C) during the first three days. He has no significant respiratory history but recently started an ACE inhibitor for hypertension. On exam, his oxygen saturation is 97% on room air, and a few bilateral basal crackles are noted.

**Questions:**

- **Q1:** If you suspect community-acquired pneumonia, what is the impact of the initial fever on this hypothesis?
- **Q2:** How does the use of an ACE inhibitor influence your hypothesis of a treatment-related cough?
- **Q3:** What is the impact of normal oxygen saturation (97%) on the probability of severe pneumonia requiring hospitalization?
- **Q4:** If you consider acute bronchitis, what is the impact of crackles on this hypothesis?
- **Q5:** How useful is the patient history in distinguishing a cough due to an ACE inhibitor from an acute respiratory infection?
- **Q6:** How does the 10-day duration of the cough influence your hypothesis of an upper respiratory tract infection?
- **Q7:** What is the importance of chest X-ray in differentiating pneumonia from bronchitis?
- **Q8:** If you suspect pneumonia, how do the initial fever and productive cough guide the need for antibiotic treatment?

Answer Choices same as Dyspnea section, with Q8 answer choices adapted to:

- Antibiotic treatment strongly indicated
- Antibiotic treatment slightly indicated
- Antibiotic treatment not influenced
- Antibiotic treatment slightly contraindicated
- Antibiotic treatment contraindicated

**Typical Example of Simulated Expert (LLM) Responses:**

**Q4: If you consider myocardial dysfunction as a possible diagnosis, how does the elevated heart rate influence this hypothesis?**
**Expert Responses:**

- **Expert 1:** Hypothesis confirmed: Tachycardia can be a compensatory response in myocardial dysfunction, increasing the likelihood of heart failure.
- **Expert 2:** Hypothesis slightly confirmed: An elevated heart rate may indicate an increased workload for the heart, often observed in myocardial dysfunction.
- **Expert 3:** Hypothesis confirmed: An elevated heart rate can be a sign of myocardial ischemia.
- **Expert 4:** Hypothesis slightly confirmed: An elevated heart rate can be a sign of myocardial dysfunction, especially in cases of acute heart failure or myocardial ischemia, which can lead to a compensatory tachycardic response. When associated with other signs such as chest tightness and acute dyspnea, this tachycardia slightly strengthens the hypothesis of myocardial dysfunction, but does not completely confirm it, as it can also be present in other acute pathologies.
- **Expert 5:** Hypothesis slightly confirmed: An elevated heart rate may be a sign of myocardial dysfunction because the heart may beat faster to compensate for reduced pumping function. This can indicate an increased workload on the heart, often seen in cases of heart failure or myocardial dysfunction.
- **Expert 6:** Hypothesis confirmed:
  1. **The tachycardia (heart rate = 110/min) fits into a very evocative clinical picture:**
     - Acute dyspnea
     - Chest tightness
     - Diaphoresis
     - Oxygen desaturation at 85%
  2. **This tachycardia is particularly significant because:**
     - It represents a typical compensatory mechanism of heart failure
     - It is accompanied by classic symptoms of myocardial dysfunction
     - The patient has cardiovascular risk factors (hypertension, smoking)
  3. **In this specific clinical context, tachycardia:**
     - Is a cardinal sign of myocardial dysfunction
     - May be both a cause and a consequence of the clinical picture
     - Fits perfectly into the scenario of acute heart failure
     - This association of signs, including tachycardia, is sufficiently characteristic to confirm (not just slightly confirm) the hypothesis of myocardial dysfunction.

**Which expert response do you find most useful?**

- Expert 1
- Expert 2
- Expert 3
- Expert 4
- Expert 5
- Expert 6

**Which expert response do you find least useful?**

- Expert 1
- Expert 2
- Expert 3
- Expert 4
- Expert 5
- Expert 6

**Was this question generated by artificial intelligence?**

- Yes
- No

**Question difficulty:**

- Very easy → 1
- ... → 5
- Very difficult → 10

**Free comments about the question:**
[Open-ended text field]
